# Active Tuberculosis Screening via a Mobile Health App in Myanmar: Incremental Cost-Effectiveness Evaluation

**DOI:** 10.2196/51998

**Published:** 2023-11-10

**Authors:** Kyaw Ko Ko Htet, Aye Nyein Phyu, Nyi Nyi Zayar, Virasakdi Chongsuvivatwong

**Affiliations:** 1 Department of Epidemiology Faculty of Medicine Prince of Songkla University Songkhla Thailand; 2 Department of Public Health National Tuberculosis Programme Ministry of Health and Sports Mandalay Myanmar

**Keywords:** mobile app, tuberculosis screening, TB screening, cost effectiveness, mHealth, mobile health, app, apps, application, applications, cost, costs, economic, economics, TB, tuberculosis, screening, communicable, x-ray, imaging, radiography, decision tree, detect, detection, detecting

## Abstract

**Background:**

A mobile app that calculates a tuberculosis (TB) risk score based on individual social and pathological characteristics has been shown to be a better predictor of the risk of contracting TB than conventionally used TB signs and symptoms (TBSS) in Myanmar, where the TB burden is high. Its cost-effectiveness, however, has not yet been assessed.

**Objective:**

This study aimed to determine the incremental costs of this mobile app and of chest x-rays (CXRs) in averting disability-adjusted life years (DALYs) among missed cases of active TB in the population being screened.

**Methods:**

Elements of incremental costs and effectiveness of 3 initial TB screening strategies were examined, including TBSS followed by CXR, the mobile app followed by CXR, and universal CXR. The incremental cost-effectiveness ratio (ICER; ie, the additional cost for each additional DALY averted) was compared to TBSS screening. Based on the latest 2020 gross domestic product (GDP) per capita of Myanmar (US $1477.50), the ICER was compared to willingness-to-pay (WTP) thresholds of 1, 2, and 3 times the GDP per capita. Probabilistic sensitivity analysis was conducted with a Monte Carlo simulation to compute the levels of probability that the ICER for each strategy was below each WTP threshold.

**Results:**

For each 100,000 population, the incremental cost compared to TBSS of active TB screening was US $345,942 for the mobile app and US $1,810,712 for universal CXR. The incremental effectiveness was 325 DALYs averted for the mobile app and 576 DALYs averted for universal CXR. For the mobile app, the estimated ICER was US $1064 (72% of GDP per capita) per 1 DALY averted. Furthermore, 100% of the simulated values were below an additional cost of 1 times the GDP per capita for 1 additional DALY averted. The universal CXR strategy has an estimated ICER of US $3143 (2.1 times the GDP per capita) per 1 DALY averted and an additional 77.2% DALYs averted compared to the app (ie, 576 – 325 / 325 DALYs); however, 0.5% of the simulated values were higher than an additional expenditure of 3 times the GDP per capita.

**Conclusions:**

Based on the status of the economy in 2020, the mobile app strategy is affordable for Myanmar. The universal CXR strategy, although it could prevent an additional 77% of DALYs, is probably unaffordable. Compared to the TBSS strategy, the mobile app system based on social and pathological characteristics of TB has potential as a TB screening tool to identify missing TB cases and to reduce TB morbidity and mortality, thereby helping to achieve the global goal of “End TB” in resource-limited settings with a high TB burden.

## Introduction

A review of national tuberculosis (TB) prevalence surveys conducted in Asia between 1990 and 2012 revealed that 40% to 60% of active TB cases are missed by routine TB signs and symptoms (TBSS) screening [1]. In Myanmar, the TB case detection rate through routine TBSS screening was reported to be 69% to 77% during the 2017 to 2019 period [2-4]. These figures highlight that nearly one-fourth of active TB cases remained undetected.

Active TB screening by chest x-ray (CXR) has been recognized as an initial screening strategy in settings with a high TB burden because of its high sensitivity and ability to detect many TB cases [5]. However, due to inadequate human resources, limited availability of CXR centers, and cost constraints, not all countries are able to implement CXR screening [6]. A previous analysis of national TB prevalence survey data showed that a propensity TB risk score derived from an individual’s social and pathological characteristics resulted in improved predictive accuracy for detecting active TB cases compared to TBSS screening [7].

A simple mobile app was previously developed to calculate the propensity TB risk score based on a provided scale [7]. The app used a cutoff score ≥0.0053 to identify presumptive TB, that is, people with ≥0.5% probability of having TB. The sensitivity of mobile app screening was 80.6% and the specificity was 63.5% [7,8]. This mobile app for community TB screening was reported to have good usability, and the individuals with presumptive TB detected by the app showed good compliance to CXR examinations [8]. However, any TB screening strategy also needs to evaluate its usability in terms of cost and effectiveness in the local context [9].

Previous research has used decision model–based analyses to assess the cost-effectiveness of TB screening in different targeted populations and with various screening options. Those studies aimed to provide evidence for policymakers and decision-makers to design large-scale trials [10,11]. In Myanmar, investigations have evaluated both the cost and effectiveness of community-based active TB case finding through TBSS [12-14]. Nevertheless, the cost-effectiveness of implementing a mobile app with a TB risk score in TB screening programs has not yet been assessed.

The aim of this study was to evaluate the incremental cost-effectiveness of active TB screening with a mobile app and CXR compared with routine passive TB screening based on TBSS in a setting with epidemiological conditions consistent with national estimates, as well as the programmatic and societal costs in the local context.

## Methods

### Study Design

This study used secondary data from the National TB Prevalence Survey of Myanmar (2010 and 2018) and data from a community-based cross-sectional TB screening survey. The community-based survey was carried out with adults aged ≥15 years in low-income urban areas of Mandalay, Myanmar, which is a densely populated city located in the central part of the country.

### Study Setting

Mandalay has 7 township health departments providing TB services. One underserved urban area was randomly selected from the list in each of 3 randomly selected townships by township medical officers and the regional TB coordinator. The prevalence of TB case notifications in Mandalay was 207 per 100,000 population during 2019 and 2020 [15].

### TB Screening Strategies

Flow charts showing TB screening strategies are shown in Multimedia Appendix 1.

#### TBSS Followed by CXR

Passive TB screening based on TBSS followed routine activities at the outpatient department (OPD) of the TB health centers [16]. Patients with TBSS seek health care at OPDs; a nurse registers them and notifies them if they have presumptive TB. A medical doctor performs a general examination and recommends that they undergo a CXR examination.

#### Mobile App Followed by CXR

The preparation phase of the mobile app screening strategy included research and development of the mobile app, consultation for sensitivity to the community with stakeholder meetings, and health staff training. The screening phase included community and household visits by health staff to identify individuals with presumptive TB via the app, upload their data for notification and registration at the corresponding TB health center, and refer them for CXR examination via a QR code referral form. The TB team leader supervised the activities at the active TB screenings.

#### Universal CXR

The preparation phase of the CXR screening strategy included consultation for sensitivity to the community with stakeholder meetings and health staff training. The screening phase included community and household visits by health staff to provide health education, identify eligible individuals aged ≥15 years within the area, and refer these individuals for CXR examination.

#### Diagnostic Procedures of TB Screening Options

Individuals with presumptive TB who accepted the CXR examination were examined with digital CXRs. Those with CXR findings suggesting TB received a Gene Xpert *Mycobacterium tuberculosis*/resistance to rifampicin (MTB/RIF) examination to detect active TB. The diagnostic activities included CXR examination by a technician and interpretation by a medical doctor, a Gene Xpert MTB/RIF examination by a microbiologist, and treatment initiation at an OPD according to national TB treatment guidelines. Differences in TB screening strategies are presented in Multimedia Appendix 2.

### Data Collection

#### Evaluation of Operational Costs of Health Care Program for TB Screening

The operational costs were evaluated using a standardized framework for cost evaluation of TB screening programs during a community-based TB screening survey [17]. Costs were evaluated for 6 major activities: preparation, screening, notification of presumptive TB at the OPD, CXRs, Gene Xpert MTB/RIF examination, and treatment initiation at the OPD. The resources used and unit cost (in US $) per participant are described in Multimedia Appendix 3. The CXR machine can perform a minimum of 12 examinations per hour, operating for 8 hours per day, thereby resulting in approximately 24,000 examinations per year. The Gene Xpert MTB/RIF machine can run 4 simultaneous tests every 2 hours, operating for 8 hours per day, which amounts to approximately 3840 tests per year. For the TBSS strategy, one CXR machine and one Gene Xpert MTB/RIF machine were used. For the mobile app strategy, 3 of each machine were used, and for the universal CXR strategy, 5 of each machine were used.

#### Human Resource Costs

The cost of human resources was calculated by multiplying the total working hours of health staff spent on TB activities by their hourly wage during the community-based TB screening survey. The hourly wage was US $0.94 for health staff and registered nurses, US $1.11 for CXR and Gene Xpert MTB/RIF technicians, and US $1.36 for medical doctors.

#### Recurrent Costs

The total costs for traveling, mobile app use, and data uploading were calculated for all participants during the community-based TB screening. The costs for a CXR referral form and digital CXRs were estimated from market-based costs obtained from the procurement section of the national TB control program conducted in 2020 under the Global Fund.

#### Capital Costs

The costs during the preparation phase were taken as capital costs for the entire program, targeting a community with a population of 100,000 to be screened [17].

The annualized capital cost of building and equipment purchases was calculated by dividing the current value of the asset by an annualizing factor, which was calculated based on the discount rate and useful life of the asset. A 3% discount rate was applied for all capital costs based on values from the Central Bank of Myanmar [18]. The useful life of a building was considered to be 50 years and for hospital equipment, 10 years, according to the Myanmar Ministry of Finance [19].

The following formula was to calculate the annualization factor [20]:

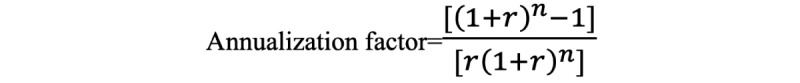



where *r* represents a 3% discount rate and *n* represents the total years of useful life.

#### Overhead Costs

Overhead costs included annual maintenance of quality control and electricity consumption (kW/h) charges for the CXR and Gene Xpert MTB/RIF machines. Annual data for the CXR and Gene Xpert MTB/RIF examinations were collected for the 2021 fiscal year (April 2020-March 2021). All local costs in Myanmar kyats were converted to US $ at an exchange rate of 1 US $= 1464 Myanmar kyats (the exchange rate on January 1, 2021).

#### Patient Costs

The patient costs included productivity loss (average US $1 per patient) and traveling to the CXR and Gene Xpert examinations (average US $0.05 per 1 kilometer) during the community-based survey.

#### Unit Cost Estimation

The unit cost for preparation for screening was calculated based on the targeted population of 100,000. The unit costs for human resources, recurrent items, and patients were computed from the total costs divided by the total number of participants in the survey. The annualized capital and overhead costs were converted into the unit cost by dividing by the total number of examinations in a year.

#### Compliance With CXR Examinations

The compliance with CXR examination was 38.2% for routine passive TB screening based on TBSS and 88% for active screening based on CXRs according to the recent 2018 National TB Prevalence Survey in Myanmar [21]. Active TB screening via the mobile health app had 71.1% CXR compliance for presumptive cases of TB detected by the app [8].

### Effectiveness Parameter

New active TB cases detected by each screening strategy, which can serve as an intermediary effectiveness parameter, were defined as individuals positive for *M tuberculosis* in the Gene Xpert MTB/RIF assay. The effectiveness of TB screening was quantified based on the number of averted disability-adjusted life years (DALYs), which are defined as the number of years lived with full health because of early detection of a new active TB case [22]. The estimated number of DALYs averted per new active TB case was 2.39 [23]. Total DALYs were calculated for each screening strategy.

### Decision Tree

The sensitivity and specificity parameters to detect active TB with TBSS, the mobile app using screening for social and pathological characteristics, CXR examinations, and the Gene Xpert MTB/RIF examinations were estimated based on analysis of the National TB Prevalence Survey [7,8]. A decision tree model was developed using those parameters based on the national TB prevalence of 468 per 100,000 population (Multimedia Appendix 4) [21]. The model populated the total number of individuals who were targeted for any of 6 major activities. The unit cost was multiplied by the total numbers to estimate the total operational costs of each TB screening strategy. The parameter estimates and unit costs are presented in Multimedia Appendices 5 and 6.

### Data Analysis

#### Incremental Cost Effectiveness of Active TB Screenings

The incremental cost effectiveness ratio (ICER), which is the additional cost for additional DALYs averted, was calculated by using the following formula:

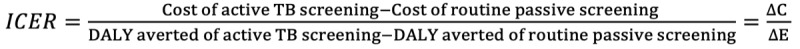



where ∆E1 and ∆C1 are the incremental cost and incremental DALYs averted from using mobile app screening, and ∆E2 and ∆C2 are the incremental cost and incremental DALYs averted from using universal CXR.

The ICERs for active screening strategies that cost less than 1 or 3 times the national annual per capita gross domestic product (GDP) per 1 DALY averted are considered highly cost-effective or cost-effective, respectively [24]. The willingness-to-pay (WTP) thresholds of the country were determined in 2020 to be below US $1477.50 (equal to 1 times the GDP per capita), US $2955 (2 times the GDP per capita), and US $4432.50 (3 times the GDP per capita) to reduce DALYs by 1 [25].

#### Probabilistic Sensitivity Analysis

Theoretically, the ICER calculated from our sample is subject to uncertainty when the result is generalized to a large population. To calculate the 95% CI of this value, it is necessary to assume a certain type of distribution for the parameters. Beta and gamma distributions were used to align with established methodologies in prior studies [10,13]. Specifically, a beta distribution was used to estimate the 95% CI for continuous random variables with a range from 0 to 1, including the prevalence of TB, sensitivity and specificity of diagnostic methods, and screening compliance assessment. Meanwhile, a gamma distribution was applied to estimate the 95% CI for parameters with nonnegative values, spanning from 0 to positive infinity, such as costs and DALYs averted [26]. Based on these distributions, probabilistic sensitivity analysis of a Monte Carlo simulation was performed for comparing the 2 screening strategies [26].

The parameters input into the Monte Carlo simulation are outlined in Multimedia Appendices 5 and 6. After conducting the simulations, 100,000 pairs of cost and effectiveness values were obtained for each comparison. These data points were plotted on a plane, with the incremental DALYs averted on the x-axis and the incremental costs on the y-axis, which we termed the cost-effectiveness plane. There are 2 sets on the plane, each of which had 100,000 points. The gray points simulate the ICER of adding universal CXR compared to TBSS screening. The black points represent screening with the mobile app compared to screening with TBSS. The diagonal lines denote the WTP thresholds of 1, 2, and 3 times GDP per capita for each DALY averted. Above the 3-times-GDP line, the ICER is beyond the WTP threshold, that is, it is not cost-effective. The proportion of points below the threshold lines is the probability of the additional effort being cost-effective. These probabilities vary by the change in the slope of the threshold lines. For example, if the WTP threshold increases, the line will become steeper and the probability of the additional effort being cost-effective will increase.

As the WTP threshold varies, the relationship between the probability of the additional efforts being cost-effective was then plotted against the WTP threshold and shown as the cost-effectiveness acceptability curve [10]. R (version 4.0.0; R Foundation for Statistical Computing) was used for data analysis and graphs.

### Ethics Approval

The study was conducted in accordance with the Declaration of Helsinki and approved by the Institutional Ethics Committee of the Faculty of Medicine, Prince of Songkla University, Hat Yai, Thailand (REC:63-074-18-1, 17-6-2020), and the Institutional Review Board of the Department of Medical Research, Myanmar (IRB00008835, 9-11-2020). As secondary data were used, the requirement for consent was waived by the ethics committee and no compensation was needed. The study data were anonymized.

## Results

### Total Operational Costs and Effectiveness of Mobile App and Universal CXR Strategies Compared to Routine TBSS Screening

The unit cost for preparation was US $0.01, while the unit cost for screening was US $0.36. In the OPD, the unit cost was US $2.74. For TBSS screening, the unit costs for CXR and Gene Xpert MTB/RIF examinations were US $6.05 and US $14.76, respectively. With the mobile app strategy, these costs were US $11.23 and US $21.23, and with the universal CXR strategy, they were US $16.42 and US $27.70, respectively.

For every 100,000 population screened, 12,578 received CXR examinations and 3757 received Gene Xpert MTB/RIF examinations for the TBSS strategy. For the mobile app strategy, these numbers were 26,098 and 8531, and for the universal CXR strategy, they were 88,000 and 18,172, respectively.

Table 1 shows the total operational costs and effectiveness of each TB screening strategy. Total costs for TB screening were 3 times higher for the mobile app strategy (US $512,214 vs US $166,272) and 11.9 times higher for the universal CXR strategy (US $1,976,984 vs US $166,272) compared to the TBSS strategy. Total DALYs averted were 2.6 times higher with the mobile app strategy (528 vs 203 DALYs averted) and 3.8 times higher with the universal CXR strategy (779 vs 203 DALYs averted) compared to the TBSS strategy.

**Table 1:** Total operational costs and effectiveness of 3 strategies: tuberculosis signs and symptoms (TBSS) followed by chest x-ray (CXR), mobile app followed by CXR, and universal CXR.

			TBSS (baseline)		Mobile app		Universal CXR
**Costs (US $)**							
	Research and development	0		263		0	
	Community sensitization with stakeholder meetings	0		612		612	
	Staff training	0		249		249	
	Screening	0		36,220		27,400	
	Notification of presumptive tuberculosis at OPD^a^	34,464		0		0	
	CXR examination^b^	76,122		293,159		1,444,520	
	Gene Xpert examination	55,453		181,105		503,310	
	Treatment initiation cost at OPD	233		606		893	
	Total	166,272		512,214		1,976,984	
**Effectiveness**							
	New tuberculosis cases detected, n	85		221		326	
	Disability-adjusted life years averted, n	203		528		779	

^a^OPD: outpatient department.

^b^This cost was calculated based on compliance with CXR examinations.

#### ICERs of the Mobile App and Universal CXR Strategies Compared to Routine TBSS Screening to Avert 1 DALY

Table 2 shows the ICERs for the mobile app and universal CXR strategies vs TBSS screening. For the mobile app, the estimated ICER was US $1064 (72% of GDP per capita) per 1 DALY averted. The universal CXR strategy had an estimated ICER of US $3143 (2.1 times GDP per capita) per 1 DALY averted.

**Table 2:** Incremental cost-effectiveness ratio of active tuberculosis (TB) screening by mobile app and chest x-ray (CXR) compared to passive TB screening by TB signs and symptoms (TBSS).

Initial Strategy	Total cost (US $)	DALYs^a^ averted, n	Difference in total cost (US $)	Difference in DALYs averted, n	US $ per DALY averted (ICER^b^)
TBSS	166,272	203	Ref	Ref	Ref
Mobile app	512,214	528	345,942	325	1064
CXR	1,976,984	779	1,810,712	576	3143

^a^DALY: disability-adjusted life year.

^b^ICER: incremental cost-effectiveness ratio.

#### Relationship Between TB Screening Costs and DALYs Averted for TBSS, Additional Use of Mobile App, and Universal CXR

**Figure 1:**
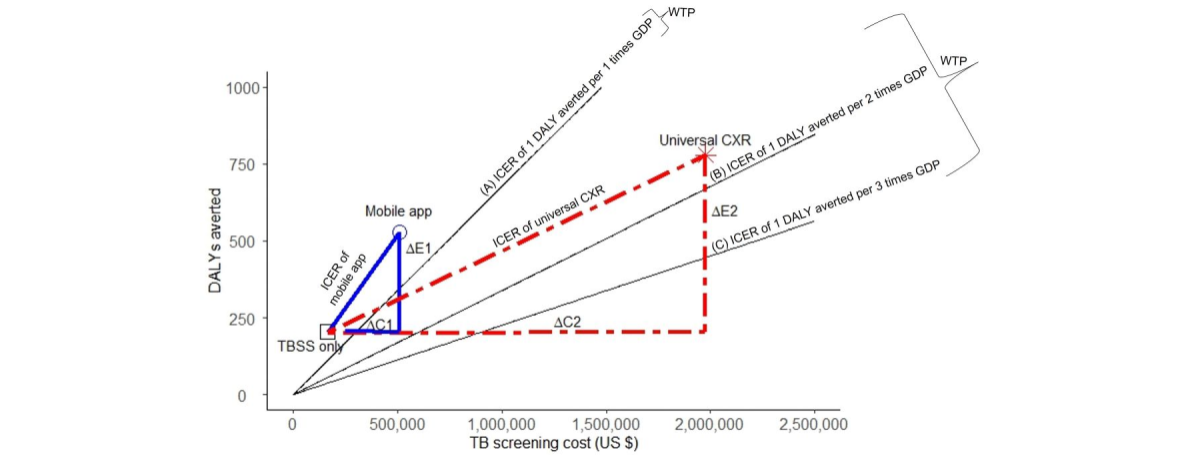
Relationship between TB screening costs and DALYs averted for TBSS (black square), additional use of the mobile app (black circle), and universal CXR (red cross). In the figure, ∆C1 represents the difference in costs between the mobile app and TBSS screening, ∆E1 represents the difference in effectiveness (DALYs averted) between the mobile app and TBSS screening, ∆C2 represents the difference in cost between universal CXR and TBSS screening, ∆E2 represents the difference in effectiveness (DALYs averted) between universal CXR and TBSS screening, ∆C1/∆E1 represents the ICER of mobile app screening, and ∆C2/∆E2 represents the ICER of universal CXR screening. A, B, and C represent the ICER of 1 DALY averted per 1, 2, and 3 times GDP per capita. CXR: chest x-ray; DALY: disability-adjusted life year; GDP: gross domestic product; TB: tuberculosis; TBSS: tuberculosis signs and symptoms; ICER: incremental cost effectiveness ratio; WTP: willingness to pay.

Figure 1 shows the relationship between the costs and DALYs averted of the 3 choices for TB screening. The additional cost of active TB screening was US $345,942 for the mobile app strategy and US $1,810,712 for the universal CXR strategy. The additional effectiveness was 325 DALYs averted with the mobile app strategy and 576 with universal CXR. Therefore, the universal CXR strategy resulted in an additional 77.2% DALYs averted compared to the app (576 – 325 / 325 DALYs averted). The TBSS screening strategy had the lowest costs and lowest DALYs averted. Universal CXR had the highest costs and DALYs averted. The mobile app was between the 2 strategies. The three sloped lined represent the WTP thresholds for 1 DALY averted per 1, 2, and 3 times GDP per capita. The slope for the ICER of mobile app screening was the steepest, indicating that it had the highest ICER below 1 times GDP per capita. The ICER slope for CXR was less steep than the ICER for the mobile app for 1 DALY averted per 1 and 2 times GDP per capita, but steeper at 1 DALY averted per 3 times GDP per capita.

### Probabilistic Sensitivity Analysis

**Figure 2:**
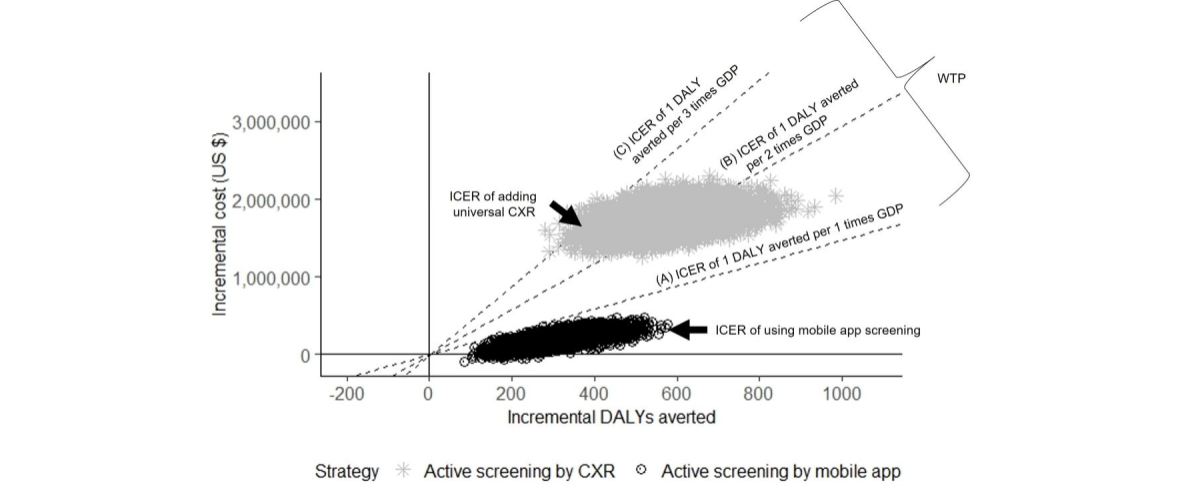
Cost-effectiveness plane of the mobile app and universal CXR strategies compared to routine TBSS screening in a probabilistic sensitivity analysis. A, B, and C represent the ICER of 1 DALY averted per 1, 2, and 3 times GDP per capita. CXR: chest x-ray; DALY: disability-adjusted life year; GDP: gross domestic product; ICER: incremental cost-effectiveness ratio; TBSS: tuberculosis signs and symptoms; WTP: willingness to pay.

**Figure 3:**
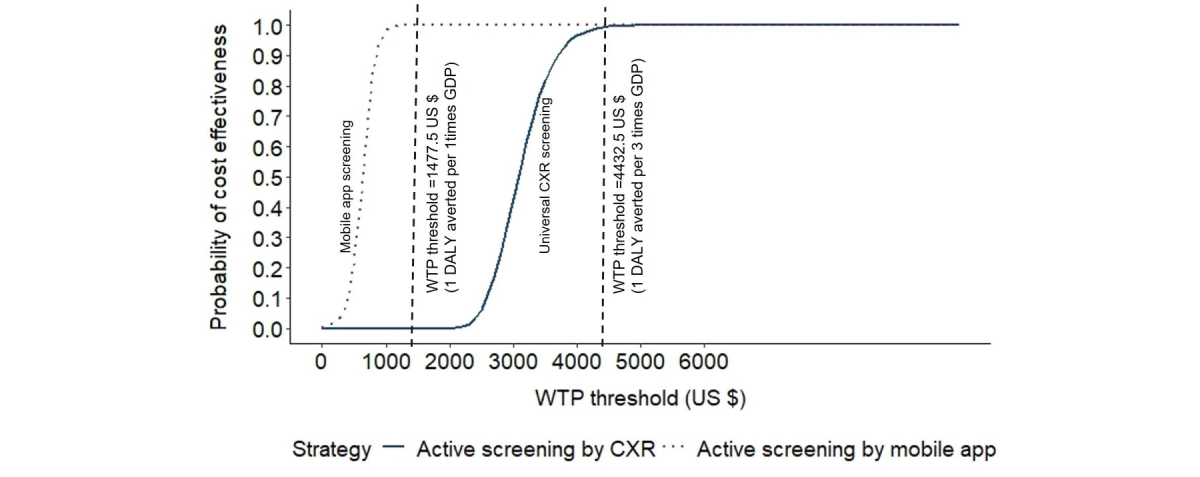
Cost-effectiveness acceptability curve of the mobile app and universal CXR strategies compared to routine TBSS screening in a probabilistic sensitivity analysis. CXR: chest x-ray; DALY: disability-adjusted life year; GDP: gross domestic product; ICER: incremental cost-effectiveness ratio; WTP: willingness to pay.

Figures 2 and 3 show the cost-effectiveness plane and acceptability curve of the mobile app strategy and universal CXR strategies compared to the TBSS screening strategy in the probabilistic sensitivity analysis.

A total of 100% of all the black simulated ICER values for using the mobile app were below the value of US $1400 (<1 times GDP per capita) to avert one DALY. Therefore, there was 100% incremental cost-effectiveness. On the other hand, 0.5% of all the gray points in universal CXR screening were beyond the WTP line of an extra 3 times GDP per capita. If the threshold lines in Figure 2 were steepened, the vertical threshold lines in Figure 3 would move to the left, indicating an increase in the probability of being cost-effective. At the threshold of more than 3 times GDP (ie, US $5000) to avert 1 DALY, the universal CXR strategy would also become incrementally cost-effective.

## Discussion

### Principal Findings

This study assessed the cost-effectiveness of active TB screening using a mobile app that uses TB risk scores based on social and pathological characteristics to identify presumptive TB cases in the community. Compared to TBSS screening, the addition of the mobile app to TB screening demonstrated cost-effectiveness, with an ICER below 1 times GDP per capita. The universal CXR strategy achieved the most DALYs averted. However, its cost was beyond 3 times GDP per capita to avert 1 DALY, much higher than TBSS.

In the “End TB” strategy, the worldwide objectives for TB control by 2035 are to achieve a 90% decrease in TB incidence and a 95% reduction in TB deaths [27]. Globally, the most recommended tools for tuberculosis screening are TBSS and CXR. TBSS screening is considered feasible at health care facilities of all levels, offering the advantage of low operational costs [28]. Nevertheless, several studies have highlighted the inadequacy of relying solely on symptom screening to combat TB and reach the global targets, primarily because of missing asymptomatic TB cases [28,29].

Like prior research, in this study, CXR screening increased TB case detection, reducing TB incidence and mortality [5,27]. However, CXR screening incurs high costs [11,27,30]. Total costs for CXR screening across the general population were more than 10 times higher than those for national TBSS screening in this study, aligning with findings in a study in Vietnam [31]. Moreover, in Myanmar, CXR facilities and diagnostic radiologists are only available at township-level hospitals, which serve approximately 150,000 to 200,000 individuals each [32]. This limited accessibility highlights the need for an efficient screening tool to refer presumptive TB cases to the health care system, a challenge faced in many countries [33] .

Community-wide active TB screening in Zambia, India, China, and South Africa has been reported to cost between US $1200 and US $9400 per DALY averted [10,34]. The estimated cost of US $3143 for the universal CXR strategy in this study is somewhat at the lower end of this range because of the high prevalence of TB in the Myanmar population. Although the ICER of the CXR strategy in this study was relatively low compared to other studies, it was still beyond 3 times GDP per capita per DALY averted, making it unaffordable. Without external financial input for the CXR strategy, the “End TB” goal to reduce TB morbidity and mortality would not be possible. Recent changes in the socioeconomic situation of the country also call for this analysis to be revisited in the near future.

### Limitations

This study did not include societal costs in the analysis. The value of DALYs averted was adapted from global estimates and might not be suitable for Myanmar. Moreover, the calculations were based on the 2020 economy of the country. The country’s GDP per capita is expected to currently be lower [25]. The shortage of health personnel should also be considered when implementing a widespread screening strategy.

### Conclusion

Based on the economy as it was in 2020, the mobile app strategy is affordable in Myanmar. The universal CXR strategy, although it can avert a high number of DALYs, is probably unaffordable. Compared to the TBSS strategy, the mobile app system of using social and pathological characteristics for TB screening is a potential TB screening tool to identify missing TB cases and reduce TB morbidity and mortality, thereby helping to achieve the global “End TB” goal in resource-limited settings with a high TB burden.

## Appendix

Multimedia Appendix 1 Flow charts of TB screening strategies. (A) Tuberculosis signs and symptoms followed by chest x-ray (CXR), (B) mobile app followed by CXR, and (C) universal CXR.

Multimedia Appendix 2 Different activities performed in tuberculosis screening strategies.

Multimedia Appendix 3 Resources used per participant.

Multimedia Appendix 4 Decision tree model to evaluate the operational costs and effectiveness of (A) tuberculosis signs and symptoms followed by chest x-ray (CXR), (B) mobile app followed by CXR, and (C) universal CXR.

Multimedia Appendix 5 Estimate and probabilistic distribution of cost parameters.

Multimedia Appendix 6 Estimates and probabilistic distribution of effectiveness parameters.

## Abbreviations

CXR: chest x-ray
DALY: disability-adjusted life years
GDP: gross domestic product
ICER: incremental cost effectiveness ratio
MTB/RIF: *Mycobacterium tuberculosis*/resistance to rifampicin
OPD: outpatient department
TB: tuberculosis
TBSS: tuberculosis signs and symptoms
WTP: willingness to pay
