# Supplementary material for: Active Tuberculosis Screening via a Mobile Health App in Myanmar: Incremental Cost-Effectiveness Evaluation
Source: JMIR Form Res. 2023 Nov 10;7:e51998. doi: 10.2196/51998 (PMC10674145; doi:10.2196/51998)
Supplement: Multimedia Appendix 1 [file formative_v7i1e51998_app1.docx]

Appendix 1 Flow charts of TB screening strategies: (A) TBSS followed by CXR, (B)mobile app followed by CXR, and (C) universal CXR

1. (C)

(B)
