# Supplementary material for: Active Tuberculosis Screening via a Mobile Health App in Myanmar: Incremental Cost-Effectiveness Evaluation
Source: JMIR Form Res. 2023 Nov 10;7:e51998. doi: 10.2196/51998 (PMC10674145; doi:10.2196/51998)
Supplement: Multimedia Appendix 2 [file formative_v7i1e51998_app2.docx]

Appendix 2 Different activities performed in TB screening strategies

| No | Activities | TB screening options | | | |
| --- | --- | --- | --- | --- | --- |
|  |  | Routine passive screening |  | Active TB  screenings | |
|  |  | TBSS |  | Mobile health app | CXR |
| 1 | **Preparation** **(P)** |  |  |  |  |
| \ | Research and development of mobile health app (P1) |  |  |  |  |
|  |  | No |  | Yes | No |
|  | Community sensitization with stakeholder meetings (P2) |  |  |  |  |
|  |  | No |  | Yes | Yes |
|  | Staff training (P3) |  |  |  |  |
|  |  | No |  | Yes | Yes |
| 2 | **Screening (S)** |  |  |  |  |
|  | Community and household visit (S1) |  |  |  |  |
|  |  | No |  | Yes | Yes |
|  | Mobile health app user fee and uploading data to notify and register at the corresponding TB health center (S2) |  |  |  |  |
|  |  | No |  | Yes | No |
|  | Supervision of TB team leader (S3) |  |  |  |  |
|  |  | No |  | Yes | Yes |
| 3 | **Notification of presumptive TB at outpatient department (O)** |  |  |  |  |
|  |  | Yes |  | No | No |
| 4 | **CXR examination (C)** |  |  |  |  |
|  |  | Yes |  | Yes | Yes |
| 5 | **Gene Xpert MTB/RIF examination (G)** |  |  |  |  |
|  |  | Yes |  | Yes | Yes |
| 6 | **Treatment initiation at outpatient department (T)** |  |  |  |  |
|  |  | Yes |  | Yes | Yes |
