# Supplementary material for: Active Tuberculosis Screening via a Mobile Health App in Myanmar: Incremental Cost-Effectiveness Evaluation
Source: JMIR Form Res. 2023 Nov 10;7:e51998. doi: 10.2196/51998 (PMC10674145; doi:10.2196/51998)
Supplement: Multimedia Appendix 3 [file formative_v7i1e51998_app3.docx]

Appendix 3 Resources used, and unit cost (USD) per participant during TB screening

| No | Cost items | Quantity  (Q) | Total resource used (km/  hour)  (TR) | Multiplier  (m) | Total cost  (TR*m or  Tc) | Unit cost per participant, (USD)  (Q/Tc) |
| --- | --- | --- | --- | --- | --- | --- |
| 1 | **Preparation (P)** |  |  |  |  |  |
|  | Research and development of mobile app (P1) |  |  |  |  |  |
|  |  |  |  |  |  | 0.00263 |
|  | Capital |  |  |  |  |  |
|  | Developing mobile app |  |  |  |  |  |
|  |  | 100000 | 120 hours | 1.36 USD per hour | 163 | 0.00163 |
|  | Cloud data storage (200 GB) |  |  |  |  |  |
|  |  | 100000 | ≠ | ≠ | 100 | 0.001 |
|  | Community sensitization with stakeholder meetings (P2) |  |  |  |  |  |
|  |  |  |  |  |  | 0.00612 |
|  | Capital |  |  |  |  |  |
|  | Meeting with three Township Medical Officers |  |  |  |  |  |
|  |  | 100000 | ≠ | ≠ | 300 | 0.003 |
|  | Meeting with three TB team leaders |  |  |  |  |  |
|  |  | 100000 | ≠ | ≠ | 300 | 0.003 |
|  | Traveling for community sensitization |  |  |  |  |  |
|  |  | 100000 | ≠ | Round trip | 12 | 0.00012 |
|  | Staff training (P3) |  |  |  |  | 0.00249 |
|  | Capital |  |  |  |  |  |
|  | Training 23 Basic Health staff |  |  |  |  |  |
|  |  | 100000 | 184 hours | USD 0.94 per hour | 172.9 | 0.0017 |
|  | Trainers |  |  |  |  |  |
|  |  | 100000 | ≠ | ≠ | 13.6 | 0.00013 |
|  | Traveling for staff training |  |  |  |  |  |
|  |  | 100000 | ≠ | Round trip | 16 | 0.00016 |
|  | Printing of training manual guideline |  |  |  |  |  |
|  |  | 100000 | ≠ | ≠ | 5 | 0.0005 |
| 2 | **Screening (S)** |  |  |  |  |  |
|  |  |  |  |  |  | 0.212 |
|  | Community and household visit by health staff (S1) |  |  |  |  |  |
|  | Human resource |  |  |  |  |  |
|  | Time spent |  |  |  |  |  |
|  |  | 631 participants approached for TB screening | 107.7 hour | USD 0.94 per hour | 101.3 | 0.160 |
|  | Recurrent |  |  |  |  |  |
|  | Traveling |  |  |  |  |  |
|  |  | 631 participants approached for TB screening | 660 km  (round trip) | USD 0.05 per 1 km | 33.03 | 0.052 |
|  | Mobile app user fee and data uploading fee to register at main server (S2) |  |  |  |  |  |
|  |  |  |  |  |  | 0.0882 |
|  | Recurrent |  |  |  |  |  |
|  |  | 631 participants approached for TB screening | ≠ | ≠ | 55.7 | 0.0882 |
|  | Supervision of TB team leader (S3) |  |  |  |  |  |
|  |  |  |  |  |  | 0.062 |
|  | Overhead |  |  |  |  |  |
|  | Supervision expenses by TB team leader |  |  |  |  |  |
|  |  | 631 participants approached for TB screening | ≠ | ≠ | 20.4 | 0.032 |
|  | Office supplies |  |  |  |  |  |
|  |  | 631 participants approached for TB screening | ≠ | ≠ | 19.35 | 0.03 |
| **3** | **Notification of presumptive TB at outpatient department (O)** |  |  |  |  |  |
|  |  |  |  |  |  | 2.74 |
|  | Human resource |  |  |  |  |  |
|  | Registration by a nurse |  |  |  |  |  |
|  |  | 631 presumptive  TB | 107.5 hour | USD 0.94 per hour | 101.2 | 0.16 |
|  | History taking and general examination by a medical doctor |  |  |  |  |  |
|  |  | 631 presumptive  TB | 269.3 hour | USD 1.36 per hour | 366.6 | 0.58 |
|  | Patient |  |  |  |  |  |
|  | Productivity loss of the presumptive TB |  |  |  |  |  |
|  |  | 631 presumptive  TB | 631 | USD 2 | 1262 | 2 |
| **4** | **CXR examination** |  |  |  |  |  |
|  |  |  |  |  |  | 6.052 |
|  | Human resource |  |  |  |  |  |
|  | Performing CXR examination by technician |  |  |  |  |  |
|  |  | 322 participants who complied to perform CXR | 40.15 hours | USD 1.11 per hour | 44.9 | 0.139 |
|  | Interpretating CXR result by medical doctor |  |  |  |  |  |
|  |  | 322 participants who complied to perform CXR | 21.3 hour | USD 1.36 per hour | 29.0 | 0.09 |
|  | Capital |  |  |  |  |  |
|  | Building |  |  |  |  |  |
|  |  | 6593 CXR examined per 2020 year | ≠ | Current value of USD 34062 with 3% discount rate per year and useful life of 50 years (Annualizing factor=25.73) | Annualized capital  cost =  1323.8 | 0.20 |
|  | Equipment purchase |  |  |  |  |  |
|  | Digital CXR  With portable generator (7.5 KVA) |  |  |  |  |  |
|  |  | 6593  CXR examined per 2020 year | ≠ | Current value of USD 104792 with 3% discount rate per year and useful life of 10 years (Annualizing factor=8.53) | Annualized capital cost=  12285 | 1.86 |
|  | Recurrent |  |  |  |  |  |
|  | CXR referral form |  |  |  |  |  |
|  |  | 322 participants who complied to perform CXR | ≠ | USD 0.02 | 6.44 | 0.02 |
|  | CXR film |  |  |  |  |  |
|  |  | 322 participants who complied to perform CXR | ≠ | USD 1.12 | 360.6 | 1.11 |
|  | Overhead |  |  |  |  |  |
|  | Maintenance for quality control |  |  |  |  |  |
|  |  | 6593 CXR examined per 2020 year | ≠ | ≠ | USD 1500 per year | 0.227 |
|  | Electricity |  |  |  |  |  |
|  |  | 6593 CXR examined per 2020 year | ≠ | ≠ | USD 500 per year | 0.075 |
|  | Patient |  |  |  |  |  |
|  | Productivity loss of the presumptive TB |  |  |  |  |  |
|  |  | 322 participants who complied to perform CXR | 322 | USD 1 | 322 | 1 |
|  | Travel to CXR center by the presumptive TB |  |  |  |  |  |
|  |  | 322 participants who complied to perform CXR | 4304.01 km | USD 0.05 per 1 km*2 for round trip | 430.4 | 1.33 |
| **5** | **Gene Xpert MTB/RIF examination** |  |  |  |  |  |
|  | Human resource |  |  |  |  |  |
|  | Performing Gene Xpert MTB/RIF test by microbiologist |  |  |  |  |  |
|  |  | 56 presumptive TB with abnormal suggestion of TB in CXR | 33.6 hour | USD 1.11 per hour | 37.62 | 0.672 |
|  | Capital |  |  |  |  |  |
|  | Building space |  |  |  |  |  |
|  |  | 1214 tested in 2020 year | ≠ | Current value of 5741 USD with 3% discount rate per year and useful life of 50 years (Annualizing factor=25.73) | Annualized capital cost=  223.1 | 0.10 |
|  | Equipment purchase |  |  |  |  |  |
|  | GeneXpert System 4-module with desktop computer |  |  |  |  |  |
|  |  | 1214 tested in 2020 year | ≠ | Current value of 17000 USD with 3% discount rate per year and useful life of 10 years  (Annualizing factor=8.53) | Annualized capital cost=  1992.9 | 1.64 |
|  | Recurrent |  |  |  |  |  |
|  | Cartridge |  |  |  |  |  |
|  |  |  | ≠ | ≠ |  | 10.28 |
|  | Overhead |  |  |  |  |  |
|  | Calibration |  |  |  |  |  |
|  |  | 1214 tested in 2020 year | ≠ | ≠ | US$ 500 per year | 0.411 |
|  | Electricity |  |  |  |  |  |
|  |  | 1214 tested in 2020 year | ≠ | ≠ | US$ 500 per year | 0.411 |
|  | Patient |  |  |  |  |  |
|  | Travel to Gene Xpert MTB/RIF center by the local health volunteer to send sputum cup of the presumptive TB |  |  |  |  |  |
|  |  | 56 presumptive TB with abnormal suggestion of TB in CXR | 698.1 km | 0.05 USD per 1 km*2 for round trip | 69.8 | 1.247 |

The CXR machine conducted a minimum of 12 examinations per hour, operating for eight hours per day, resulting in approximately 24,000 examinations per year. The Gene Xpert MTB/RIF machine ran four simultaneous tests every two hours, operating for eight hours per day, which amounted to approximately 3,840 tests per year. For the TBSS strategy, one CXR machine and one Gene Xpert MTB/RIF machine were used. For the mobile app strategy, three of each machine were employed, and for the CXR-for-all strategy, five of each machine were utilized.

**Unit costs of CXR examination for each strategy**

Unit cost of CXR examination for TBSS strategy (One CXR machine is required)

= (1 x Human resource) + (1 x Capital cost) + Recurrent cost + (1 x Overhead cost) + Patient cost

= (1 x 0.229) + (1 x 2.06) + 1.13 + (1 x 0.302) + 2.33

= 6.052 USD

Unit cost of CXR examination for mobile app strategy (Three CXR machines are required)

= (3 x Human resource) + (3 x Capital cost) + Recurrent cost + (3 x Overhead cost) + Patient cost

=(3 x 0.229) + (3 x 2.06) + 1.13 + (3 x 0.302) + 2.33

= 11.233 USD

Unit cost of CXR examination for CXR for all strategy (Five CXR machines are required)

= (5 x Human resource) + (5 x Capital cost) + Recurrent cost + (5 x Overhead cost) + Patient cost

=(5 x 0.229) + (5 x 2.06) + 1.13 + (5 x 0.302) + 2.33

= 16.415 USD

**Unit cost of Gene Xpert MTB.RIF examination for each strategy**

Unit cost of Gene Xpert MTB/RIF examination for TBSS strategy (One machine is required)

= (1 x Human resource) + (1 x Capital cost) + Recurrent cost + (1 x Overhead cost) + Patient cost

=(1 x 0.672) + (1 x 1.74) + 10.28 + (1 x 0.822) + 1.247

= 14.76

Unit cost of Gene Xpert MTB/RIF examination for mobile app strategy (Three machines are required)

= (3 x Human resource) + (3 x Capital cost) + Recurrent cost + (3 x Overhead cost) + Patient cost

=(3 x 0.672) + (3 x 1.74) + 10.28 + (3 x 0.822) + 1.247

=21.229 USD

Unit cost of Gene Xpert MTB/RIF examination for CXR for all strategy (Five machines are required)

= (5 x Human resource) + (5 x Capital cost) + Recurrent cost + (5 x Overhead cost) + Patient cost

=(5 x 0.672) + (5 x 1.74) + 10.28 + (5 x 0.822) + 1.247

=27.697 USD
