# Supplementary material for: Active Tuberculosis Screening via a Mobile Health App in Myanmar: Incremental Cost-Effectiveness Evaluation
Source: JMIR Form Res. 2023 Nov 10;7:e51998. doi: 10.2196/51998 (PMC10674145; doi:10.2196/51998)
Supplement: Multimedia Appendix 4 [file formative_v7i1e51998_app4.docx]

Appendix 4 Decision tree model to evaluate the operational costs and effectiveness of (A) TBSS followed by CXR, (B) mobile app followed by CXR and (C) universal CXR

(A)


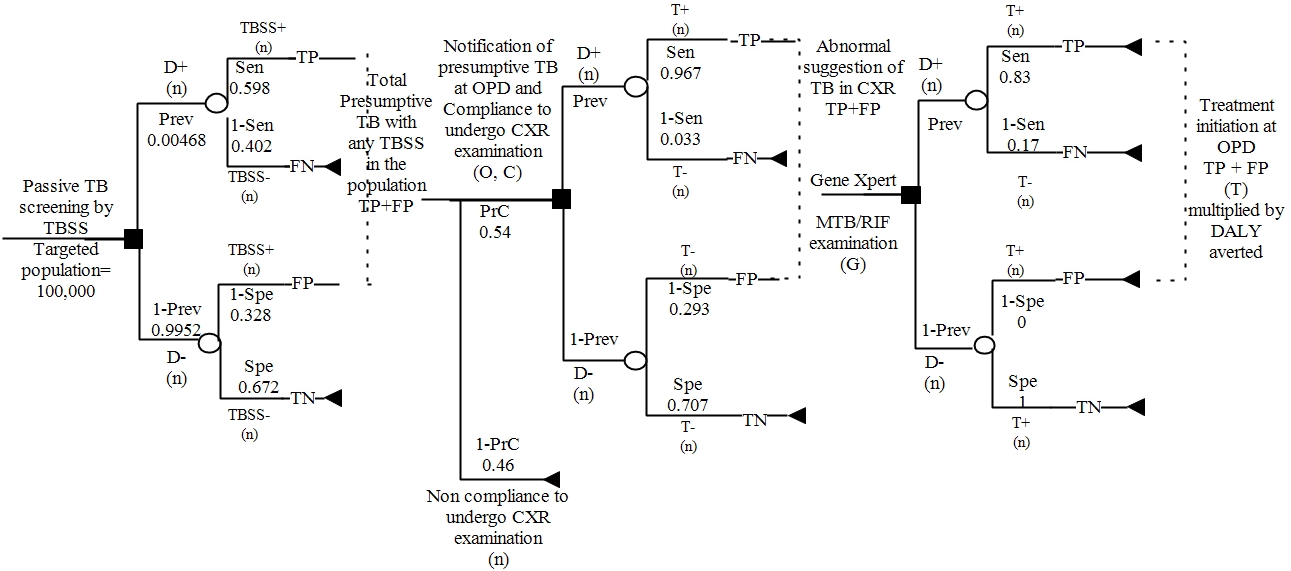


(B)


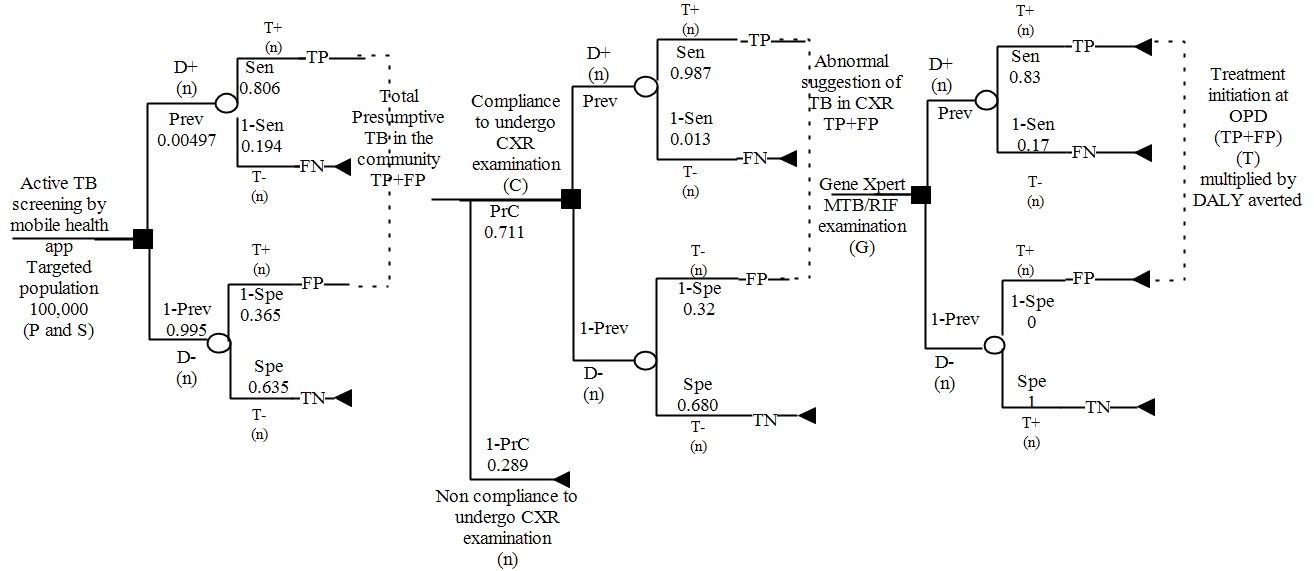


(C)


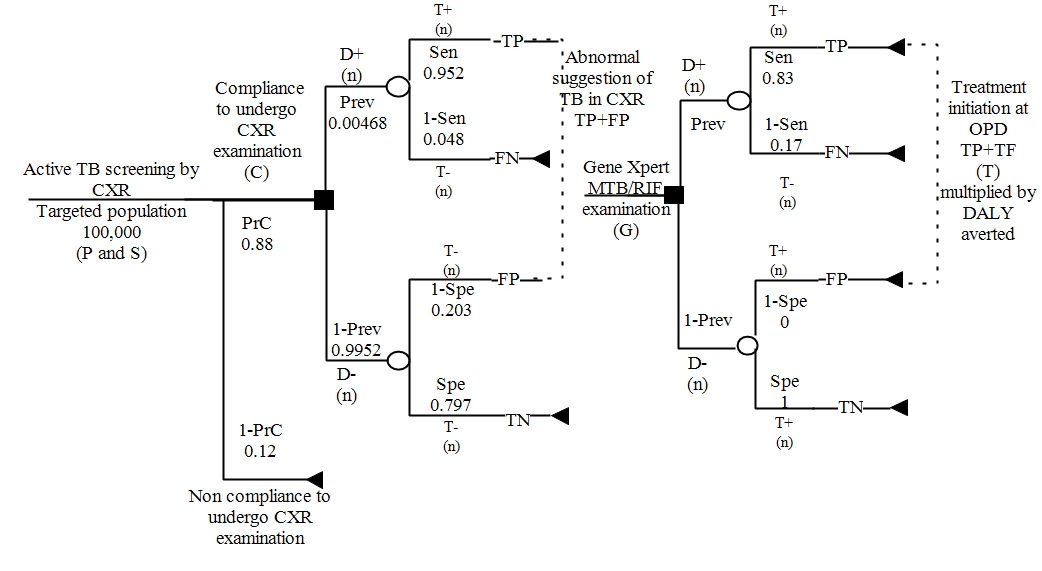


CXR=Chest x-ray, D+=Disease positive, D-=Disease negative, FN=False Negative, FP=False positive, PrC=Proportion of compliance for each test, 1-PrC=Proportion of non-compliance for each test, Prev=Prevalence of disease, Sen=Sensitivity of a test, 1-Sen=1-Sensivity of a test, Spe=Specificity of a test, 1-Spe=1-Specificity of a test, T+=Test positive, T-=Test negative, TP= True positive, TN=True negative, Prev=TP divided by sum of TP and TF,n=number of disease positive or test positive, DALY averted=Disability adjusted life years averted of a new TB case deteced

Note: P,S,O,C,G and T denotes total number of participants involved in preparation, screening, notification of presumptive TB at outpatient department, CXR, Gene Xpert MTB/RIF examinations, treatment registration and initiation, respectively which are multipled by unit costs to calcualte the total operational costs.

TBSS=Tuberculosis signs and symptoms, OPD=Outpatient department
