# Supplementary material for: Active Tuberculosis Screening via a Mobile Health App in Myanmar: Incremental Cost-Effectiveness Evaluation
Source: JMIR Form Res. 2023 Nov 10;7:e51998. doi: 10.2196/51998 (PMC10674145; doi:10.2196/51998)
Supplement: Multimedia Appendix 5 [file formative_v7i1e51998_app5.docx]

Appendix 5 Estimate and probabilistic distribution of cost parameters

| Activities | Estimates  Mean (Standard deviation) | Distribution | k | θ | Range | 95% CI | |
| --- | --- | --- | --- | --- | --- | --- | --- |
|  |  |  |  |  |  | Low | High |
| **Preparation (P)** |  |  |  |  |  |  |  |
| Research and development of mobile health app (P1) |  |  |  |  |  |  |  |
|  | 0.00263 (0.0013) | Gamma | 4 | 0.0006575 | 0.0-0.012 | 0.0007 | 0.0057 |
| Community sensitization with stakeholder meetings (P2) |  |  |  |  |  |  |  |
|  | 0.00612 (0.00306) | Gamma | 4 | 0.00153 | 0.0-0.027 | 0.0016 | 0.013 |
| Staff training (P3) |  |  |  |  |  |  |  |
|  | 0.00249 (0.0012) | Gamma | 4 | 0.0006225 | 0.0-0.01 | 0.0006 | 0.0053 |
| **Screening (S)** |  |  |  |  |  |  |  |
| Community and household visit (S1) |  |  |  |  |  |  |  |
|  | 0.212 (0.106) | Gamma | 4 | 0.053 | 0.012-1.276 | 0.059 | 0.467 |
| Mobile health app user fee and uploading data to notify and register at the corresponding TB health center (S2) |  |  |  |  |  |  |  |
|  | 0.0882 (0.00441) | Gamma | 4 | 0.002205 | 0.001-0.035 | 0.0023 | 0.0194 |
| Supervision of TB team leader (S3) |  |  |  |  |  |  |  |
|  | 0.062 (0.031) | Gamma | 4 | 0.0155 | 0.004-0.255 | 0.016 | 0.134 |
| **Notification of presumptive TB at outpatient department (O)** |  |  |  |  |  |  |  |
|  | 2.74 (1.06) | Gamma | 6.681737 | 0.410073 | 0.332-8.42 | 1.077 | 5.107 |
| **CXR examination (C)** |  |  |  |  |  |  |  |
|  | 6.052 (1.18) | Gamma | 26.30473 | 0.2300727 | 2.67-12.06 | 3.994 | 8.536 |
| **Gene Xpert MTB/RIF examination (G)** |  |  |  |  |  |  |  |
|  | 14.76 (0.46) | Gamma | 1029.712 | 0.01433507 | 13.11-  16.64 | 13.869 | 15.702 |
|  |  |  |  |  |  |  |  |
| **Treatment initiation at outpatient department (T)** |  |  |  |  |  |  |  |
|  | 2.74 (1.06) | Gamma | 6.681737 | 0.410073 | 0.332-8.42 | 1.077 | 5.107 |

Range* was calculated from probabilistic gamma distribution.
