# Supplementary material for: Active Tuberculosis Screening via a Mobile Health App in Myanmar: Incremental Cost-Effectiveness Evaluation
Source: JMIR Form Res. 2023 Nov 10;7:e51998. doi: 10.2196/51998 (PMC10674145; doi:10.2196/51998)
Supplement: Multimedia Appendix 6 [file formative_v7i1e51998_app6.docx]

Appendix 6 Estimates and probabilistic distribution of effectiveness parameters

| Parameters | Estimates | Distribution | α | β | Range | 95% CI | | Reference |
| --- | --- | --- | --- | --- | --- | --- | --- | --- |
|  |  |  |  |  |  | Low | High |  |
| **Prevalence of TB** |  |  |  |  |  |  |  |  |
|  | 0.00468 | Beta | 467.9953 | 99531 | 0.004-0.006 | 0.0042 | 0.0051 | [21] |
| **(A) Routine passive screening by TBSS** |  |  |  |  |  |  |  |  |
| **TBSS screening** |  |  |  |  |  |  |  |  |
| Sensitivity |  |  |  |  |  |  |  |  |
|  | 0.598 | Beta | 59799.4 | 40199.6 | 0.591-0.605 | 0.594 | 0.601 | [7] |
| Specificity |  |  |  |  |  |  |  |  |
|  | 0.672 | Beta | 67199.33 | 32799.67 | 0.666-0.679 | 0.669 | 0.674 | [7] |
| **Proportion of compliance to undergo CXR examination among the presumptive TB with TBSS** |  |  |  |  |  |  |  |  |
|  | 0.382 | Beta | 8.636103 | 13.9715 | 0.057-0.804 | 0.198 | 0.585 | [21] |
| **CXR examination** |  |  |  |  |  |  |  |  |
| Sensitivity |  |  |  |  |  |  |  |  |
|  | 0.967 | Beta | 96699.03 | 3299.967 | 0.965-0.969 | 0.965 | 0.968 | [7] |
| Specificity |  |  |  |  |  |  |  |  |
|  | 0.707 | Beta | 70699.29 | 29299.71 | 0.701-0.714 | 0.704 | 0.709 | [7] |
| **Gene Xpert MTB/RIF examination** |  |  |  |  |  |  |  |  |
| Sensitivity |  |  |  |  |  |  |  |  |
|  | 0.83 | Beta | 82999.17 | 16999.83 | 0.825-0.835 | 0.827 | 0.832 | [7] |
| Specificity |  |  |  |  |  |  |  |  |
|  | 1 |  |  |  | 1 |  |  | [7] |
|  |  |  |  |  |  |  |  |  |
| **(B) Active TB screening by mobile app** |  |  |  |  |  |  |  |  |
| **Mobile app screening** |  |  |  |  |  |  |  |  |
| Sensitivity |  |  |  |  |  |  |  |  |
|  | 0.806 | Beta | 80599.19 | 19399.81 | 0.80-0.812 | 0.803 | 0.808 | [8] |
| Specificity |  |  |  |  |  |  |  |  |
|  | 0.635 | Beta | 63499.36 | 36499.64 | 0.628-0.642 | 0.632 | 0.637 | [8] |
| **Proportion of compliance to undergo CXR examination by presumptive TB detected by the app** |  |  |  |  |  |  |  |  |
|  | 0.711 | Beta | 145.3846 | 59.09443 | 0.56-0.831 | 0.646 | 0.771 | [8] |
| **CXR examination** |  |  |  |  |  |  |  |  |
| Sensitivity |  |  |  |  |  |  |  |  |
|  | 0.987 | Beta | 98699.01 | 1299.987 | 0.985-0.989 | 0.986 | 0.987 | [8] |
| Specificity |  |  |  |  |  |  |  |  |
|  | 0.680 | Beta | 67999.32 | 31999.68 | 0.674-0.686 | 0.677 | 0.682 | [8] |
| **Gene Xpert MTB/RIF examination** |  |  |  |  |  |  |  |  |
| Sensitivity |  |  |  |  |  |  |  |  |
|  | 0.83 | Beta | 82999.17 | 16999.83 | 0.82-0.835 | 0.827 | 0.832 | [7] |
| Specificity |  |  |  |  |  |  |  |  |
|  | 1 |  |  |  | 1 |  |  | [7] |
| **(C) Active TB screening by CXR** |  |  |  |  |  |  |  |  |
| **Proportion of compliance to undergo CXR examination among the general population** |  |  |  |  |  |  |  |  |
|  | 0.88 | Beta | 92.048 | 12.552 | 0.69-0.97 | 0.811 | 0.934 | [21] |
| **CXR examination** |  |  |  |  |  |  |  |  |
| Sensitivity |  |  |  |  |  |  |  |  |
|  | 0.952 | Beta | 95199.05 | 4799.952 | 0.949-0.955 | 0.950 | 0.953 | [7] |
| Specificity |  |  |  |  |  |  |  |  |
|  | 0.797 | Beta | 79699.2 | 20299.8 | 0.791-0.803 | 0.794 | 0.799 | [7] |
| **Gene Xpert MTB/RIF examination** |  |  |  |  |  |  |  |  |
| Sensitivity |  |  |  |  |  |  |  |  |
|  | 0.83 | Beta | 82999.17 | 16999.83 | 0.82-0.835 | 0.827 | 0.832 | [7] |
| Specificity |  |  |  |  |  |  |  |  |
|  | 1 |  |  |  | 1 |  |  | [7] |

Range* was calculated from probabilistic beta distribution.
